# Patient-Reported Outcomes Measurement Information System (PROMIS) instruments among individuals with symptomatic knee osteoarthritis: a cross-sectional study of floor/ceiling effects and construct validity

**DOI:** 10.1186/s12891-015-0715-y

**Published:** 2015-09-14

**Authors:** Jeffrey B. Driban, Nani Morgan, Lori Lyn Price, Karon F. Cook, Chenchen Wang

**Affiliations:** Division of Rheumatology, Tufts Medical Center, 800 Washington Street, Box #406, Boston, MA 02111 USA; Internal Medicine Residency Program, John A Burns School of Medicine, University of Hawaii, Honolulu, HI USA; The Institute for Clinical Research and Health Policy Studies, Tufts Medical Center, and Tufts Clinical and Translational Science Institute, Tufts University, 800 Washington Street, Box #63, Boston, MA 02111 USA; Department of Medical Social Sciences, Feinberg School of Medicine, Northwestern University, Chicago, IL USA

## Abstract

**Background:**

The psychometric properties of Patient Reported Outcomes Measurement Information System (PROMIS) instruments have been explored in a number of general and clinical samples. No study, however, has evaluated the psychometric function of these measures in individuals with symptomatic knee osteoarthritis (KOA). The aim of this project was to evaluate the construct (structural) validity and floor/ceiling effects of four PROMIS measures in this population.

**Methods:**

We conducted a secondary analysis of baseline data from a randomized trial comparing Tai Chi and physical therapy. Participants completed four PROMIS static short-form instruments (i.e., Anxiety, Depression, Physical Function, and Pain Interference) as well as six well-validated (legacy) measures that assess pain, function, and psychological health. We calculated descriptive statistics and percentages of participants scoring the minimum (floor) and maximum (ceiling) possible scores for PROMIS and legacy measures. We also estimated the association between PROMIS scores and scores on legacy measures using Spearman’s rank correlations coefficients.

**Results:**

Data from 204 participants were analyzed. Mean age of the sample was 60 years; 70 % were female. The PROMIS Anxiety and Depression had floor effects with 17 and 24 % of participants scoring the minimum, respectively. PROMIS Anxiety and Depression scores had strongest associations with general mental health, including stress (Perceived Stress Scale, *r* ≥ 0.65) and depression (Beck Depression Index-II, *r* = 0.70). PROMIS Pain Interference scores correlated most strongly with measures of whole body pain (Short-Form 36 Bodily Pain, *r* = −0.73) and physical health (Short-Form 36 Physical-Component Summary, *r* = −0.73); their correlations were lower with other legacy measures, including with the WOMAC knee-specific pain (*r* = 0.47). PROMIS Physical Function scores had stronger associations with scores on the Short-Form 36 Physical Function (*r* = 0.79) than with scores on other legacy measures.

**Conclusion:**

The four PROMIS static-short forms performed well among individuals with symptomatic knee osteoarthritis as evidenced in correlations with legacy measures. PROMIS Anxiety and Depression target general mental health (e.g., stress, depression), and PROMIS Pain Interference and Physical Function static-short forms target whole-body outcomes among participants with symptomatic knee osteoarthritis. Floor effects in the PROMIS Anxiety and Depression scores should be considered if needing to distinguish among patients with very low levels of these outcomes.

**Trial registration:**

Clinicaltrials.gov NCT01258985. Registered 10 December 2010

**Electronic supplementary material:**

The online version of this article (doi:10.1186/s12891-015-0715-y) contains supplementary material, which is available to authorized users.

## Background

Osteoarthritis is a disease characterized by structural changes throughout the joint but it is also an illness of the whole patient defined by patient-reported outcomes such as pain, stiffness, and disability [1]. Furthermore, a patient with osteoarthritis often participates less in valued activities and may experience fatigue as well as disrupted mood, sleep, and quality of life [2–4]. Therefore, we must measure a wide array of patient-reported outcomes to thoroughly assess health status and treatment efficacy among individuals with osteoarthritis.

There are numerous patient-reported outcome measures to assess individuals with osteoarthritis. Excessive proliferation of these measures and lack of standardization in their use, however, hinder comparisons across studies and populations [5]. Furthermore, using multiple measures may be time consuming for patients, cost prohibitive, and not practical for all settings. The National Institutes of Health’s (NIH) Patient Reported Outcomes Measurement Information System (PROMIS) has introduced a number of static short-form patient-reported outcome measures, which offer an efficient and cost-effective alternative [6]. PROMIS is unique because it is intended for clinicians and researchers in various disciplines who are interested in measuring physical, mental, and social health among individuals with various chronic conditions. Several static-short form instruments target constructs relevant to patients with symptomatic knee osteoarthritis, including PROMIS Pain Interference 6b, Physical Function 10a, Emotional Distress-Anxiety 7a, and Emotional Distress-Depression 8b. Evidence for the validity of these instruments was initially obtained based on analysis of responses from a general population sample and from clinical samples, including a subset with osteoarthritis [7]. However, no prior study has evaluated the properties of PROMIS static short-form scores among individuals with symptomatic knee osteoarthritis.

Our objective was to evaluate the floor/ceiling effects and construct (structural) validity of four PROMIS Short-Form Instruments (i.e., Pain Interference 6b, Physical Function 10a, Emotional Distress-Anxiety 7a, and Emotional Distress-Depression 8b) function among a well-characterized sample of individuals with knee osteoarthritis. To achieve our goals we asked participants to complete six legacy measures: Medical Outcomes Short Form-36 (SF-36) [8–10], the Western Ontario and McMaster Universities Arthritis Index (WOMAC) [11–13], two objective physical function tests (i.e., 6-min walk test, 20-meter walk test) [14], the Perceived Stress Scale [15], and the Beck Depression Inventory Second Edition (BDI-II) [16–19]. These measures of pain, function, and psychological health domains were chosen because they relate to the constructs measured by the four PROMIS static short-form instruments and are widely used and well-validated among individuals with knee osteoarthritis.

## Methods

We conducted a secondary analysis of baseline data obtained in our NIH-funded, randomized trial that compared Tai Chi and physical therapy among individuals with symptomatic knee osteoarthritis (Trial Registry #NCT01258985) [20]. This secondary dataset is unique because it includes responses to a wide range of patient-reported outcome measures, both PROMIS measures and well-validated (legacy) measures used widely in studies of knee osteoarthritis. Data were collected at Tufts Medical Center, an urban tertiary care academic hospital in Boston, USA. We received ethics approval for this study from the Tufts Medical Center/ Tufts University Human Institutional Review Board. To be eligible, participants had to meet the following criteria: 1) age ≥40 years; 2) Western Ontario and McMaster Universities Arthritis Index (WOMAC) pain subscale score (100 mm visual analog scales) >40 on at least 1 out of 5 questions; 3) fulfillment of the American College of Rheumatology criteria for knee osteoarthritis [21]; 4) radiographic evidence of knee osteoarthritis defined as the presence of osteophytes in the tibiofemoral and/or the patellofemoral compartment, as assessed on standing anterior-posterior and lateral views; and 5) confirmation of knee pain, discomfort, or disability by clinical examination. We excluded individuals who had experience in the past year with physical therapy, Tai Chi training, or similar types of alternative medicine (e.g., Qi Gong or yoga); serious medical conditions limiting their ability to fully participate as determined by a primary care physician; intra-articular steroid injections or replacement surgery on the affected knee in the previous three months; or a Mini-Mental examination score <24. All participants enrolled in the study provided informed consent. For this secondary analysis, we included all 204 participants who had their baseline visits between March 2011 and June 2013. These 204 participants were selected after staff prescreened almost 1,200 individuals on the phone and then screened approximately 280 individuals in person. The most common reason a person was not randomized into the study was if the individual failed to meet the inclusion criteria or an individual declined to participate.

### PROMIS instruments

Participants enrolled in the trial completed four PROMIS static short-form, version 1.0 instruments including PROMIS Pain Interference 6b, Physical Function 10a, Emotional Distress-Anxiety 7a, and Emotional Distress-Depression 8b. These PROMIS instruments were the original versions of the short forms. The instruments were selected to meet the goal of the parent study: to compare the influence of Tai Chi or physical therapy on physical and psychological outcome measures.

The validity of PROMIS instruments has been assessed in both general and clinical U.S. sample populations [7, 22–24]. PROMIS short forms represent the items of the PROMIS item banks constructed to measure the targeted constructs. All of the included PROMIS short forms used 5-point Likert-type response categories to capture intensity, frequency, or duration, described in detail below. The instruments use a seven-day recall period, with the exception of PROMIS Physical Function, which does not reference any timeframe.

PROMIS instruments are publicly available on the PROMIS Assessment Center Library website: http://www.assessmentcenter.net/PromisForms.aspx. Sample questions are available at http://www.nihpromis.org/measures/SampleQuestions. Scoring manuals for PROMIS measures (http://www.assessmentcenter.net/Manuals.aspx) outline the development of the short forms (also see http://www.nihpromis.org/science/sciencehome), report psychometric properties for each instrument, and describe how to identify PROMIS T-scores based on short form raw summed item scores. For all of the PROMIS short forms we reported the PROMIS T-scores.

PROMIS Short Form v1.0 - Pain Interference 6b measures “impact of pain on physical, mental, and social activities” (6). It consists of 6 items measured on a 5-point Likert-type scale (first 5 questions: “not at all” to “very much”; final question: “never” to “always”). The questions assess the degree to which pain interferes with enjoyment in life, ability to concentrate, day-to-day activities, recreational activities, tasks away from home, and socializing with others. The scores range from 41 to 78.3 with higher scores representing worse pain impact.

PROMIS Short Form v1.0 - Physical Function 10a measures “ability to carry out various activities that require physical capability, ranging from self-care to more vigorous activities that require increasing degrees of mobility, strength, or endurance” [7]. Half the 10 items of this form address the degree to which the respondent’s health limits physical activities such as carrying groceries, climbing stairs, or participating in sports (“not at all” to “cannot do”). The other five items address the level of difficulty faced in carrying out activities of daily living such as vacuuming or tying one’s shoelace (“without any difficulty” to “unable to do”). The scores range from 14.1 to 61.7 with higher scores representing better functioning.

PROMIS Short Form v1.0 - Emotional Distress- Anxiety 7a measures “fear, anxious misery, hyperarousal, and somatic symptoms related to arousal” [7]. The instrument consists of 7 items that ask respondents about the frequency with which they experienced emotions such as fear, stress, and anxiety (“never” to “always”). Scores range from 36.3 to 82.7 with higher scores indicating worse anxiety.

PROMIS Short Form v1.0 - Emotional Distress - Depression 8b measures “negative mood, decrease in positive affect, information processing deficits, negative views of the self, and negative social cognition” [7]. It consists of 8 items in which respondents indicate the frequency with which they have experienced emotions such as worthlessness, hopelessness, and sadness (“never” to “always”). Scores range from 35.2 to 82.4 with higher scores representing worse depression.

### Legacy measures

The SF-36 is a measure of general health status comprised of eight scales: physical functioning, social functioning, bodily pain, energy and vitality, mental health, role limitations due to physical problems, role limitations due to emotional problems, and general health. The eight scales are summarized in the Physical Component Summary (PCS) and Mental Component Summary (MCS) scores. The SF-36 uses a 4-week recall period and consists of 36 items. Scores range from 0 to 100 with higher scores indicating better health. The SF-36 has been used widely in clinical osteoarthritis trials and the validity of SF-36 scores as a measure of health status has been well documented [8–10].

The WOMAC is a disease-specific measure designed to evaluate joint-specific health status and health outcomes in knee and hip osteoarthritis. We used the form consisting of 100 mm horizontal visual analogue scales with a 48-h recall period. It has three subscales: pain (score range, 0–500), stiffness (0–200), and function (0–1700) with higher subscale scores indicating more severe disease. The validity of WOMAC has been well-established in knee and hip osteoarthritis [11–13].

The 6-min walk test and 20-m walk test are performance-based measures of gait velocity used to assess lower extremity function and mobility. The 6-min walk test is a measure of distance walked (measured in meters), while walking as fast as possible, in a total of six minutes, with greater distances indicating higher capacity. The 20-m walk test is a measure of time (measured in seconds) required to walk a total of twenty meters, with longer times indicating lower capacity. Both tests were performed in quiet hallways and were administered by trained investigators following a standard script. Gait velocity measures are valid for use in knee osteoarthritis populations [14].

The Perceived Stress Scale is a measure of non-specific stress appraisal. It uses a 1-month recall period and consists of 10 items on a 5-point Likert scale. Scores range from 0 to 40 with higher scores indicating higher levels of experienced stress. The validity of Perceived Stress Scale scores has been well documented in knee osteoarthritis studies [15].

The BDI-II is a measure of depression that assesses commonly associated signs and symptoms according the Diagnostic and Statistical Manual of Mental Disorders, Fourth Edition criteria. It uses a 2-week recall period and consists of 21 multiple-choice items. Scores range from 0 to 63 with higher scores indicating more severe depression. The BDI-II has been validated for use among non-psychiatric populations and has been used to assess depression in multiple knee osteoarthritis studies [16–19].

### Procedure

As part of their baseline assessment and prior to any intervention, participants completed the outcome measures described above. The outcome measures were completed in the following order: WOMAC, SF-36, Perceived Stress Scale, BDI-II, PROMIS Pain Interference, PROMIS Physical Function, PROMIS Depression, and PROMIS Anxiety. All of the self-reported outcome measures were collected and managed using REDCap electronic data capture tools [25] hosted at Tufts Medical Center except for WOMAC, which was completed on paper. The two physical function tests were completed on site and were administered either prior to or after completion of the questionnaires, depending on the availability of research staff.

### Statistical analysis

The analyses reported here was limited to baseline data obtained from participants that met eligibility criteria and were enrolled in the study. We calculated descriptive statistics and percentages of participants scoring the minimum (floor) and maximum (ceiling) possible scores. We defined important floor or ceiling effects as more than 15 % of participants achieving the lowest or highest score, respectively [26]. Because not all data were normally distributed, we estimated the association between scores on the PROMIS instruments and legacy measures using the non-parametric, Spearman’s rank correlation coefficient.

### Comparison of results with previous literature

Tests of the statistical significance of differences in associations were outside the scope of this study and observed differences in point estimates of correlations should not be over-interpreted. However, to provide an interpretive context for our results, we presented the current results in the context of previous correlational studies. We conducted a comprehensive literature search (in Summer 2014) for articles that reported correlations between scores from legacy measures and PROMIS scores or other instruments that measure similar constructs. Results gleaned from published literature were grouped by clinical status: general population, osteoarthritis, other clinical conditions, and the current study results. Findings were plotted by condition on a graph to visually represent the range of findings in previous studies.

## Results

### Demographic data and descriptive statistics

Our analysis included data from 204 participants with an average age of 60 years and body mass index of 33 kg/m^2^ (Table 1). Ninety-two percent had a Kellgren/Lawrence grade ≥ 2 in the tibiofemoral joint with a median self-reported duration of knee pain of 5 years. Table 1 includes additional demographic and clinical characteristics for the sample.

**Table 1:** Demographic and clinical characteristics of patients with knee osteoarthritis (*n* = 204)

Characteristic	Distribution n (%)^a^
Age, mean ± SD years	60.2 (10.5)
Body Mass Index, mean ± SD kg/m^2^	32.8 (7.2)
Self-reported duration of knee pain, median (25^th^, 75^th^ percentile) years	5.0 (3.0, 10.0)
Tibiofemoral Kellgren/Lawrence grade ≥ 2	183 (92.4)
Female Sex	143 (70.1)
Race	
White	107 (52.7)
Black	72 (35.5)
Other	24 (11.8)
Married	52 (25.5)
Greater than high school education	170 (83.3)

^a^n (%) unless noted otherwise. SD = standard deviation

**Fig. 1:**
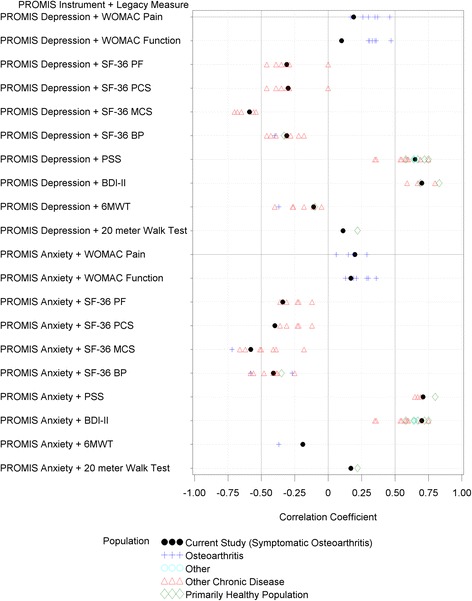
Correlations between Anxiety and Depression and Legacy Measures. References for the correlations are provided in the supplementary file. To develop a reference set of correlations we considered correlations between scores from legacy measures and PROMIS scores or other instruments that measure similar constructs. SF-36 = short form 36, PF = physical function, PCS = physical component score, MCS = mental component score, BP = bodily pain, PSS = Perceived Stress Scale, BDI-II = Beck Depression Inventory – II, and 6MWT = 6-min walk test

**Fig. 2:**
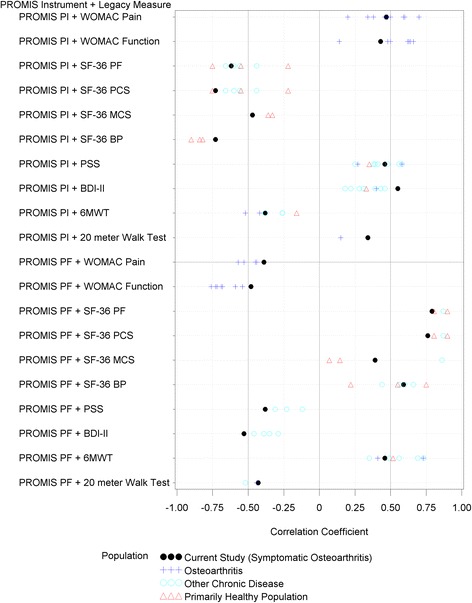
Correlations between Pain Interference (PI) and Physical Function (PF) and Legacy Measures. References for the correlations are provided in the supplementary file. To develop a reference set of correlations we considered correlations between scores from legacy measures and PROMIS scores or other instruments that measure similar constructs. SF-36 = short form 36, PF = physical function, PCS = physical component score, MCS = mental component score, BP = bodily pain, PSS = Perceived Stress Scale, BDI-II = Beck Depression Inventory – II, and 6MWT = 6-min walk test

Floor or ceiling effects are reported in Table 2. Additional file 1: Table S1 and Figs. 1 and 2 report the correlation coefficients between PROMIS static short-form instruments and legacy measures.

**Table 2:** Descriptive statistics of outcome measures in patients with knee osteoarthritis including floor and ceiling effects

Outcome Measure	Scores				Floor		Ceiling	
	Score Range	*n*	Mean ± SD	Median (range)	*n*	%	*n*	%
PROMIS Pain Interference	41-78.3	204	58.0 (7.0)	58.1 (41.0-74.4)	11	5.4	0	0
PROMIS Physical Function^a^	14.1-61.7	204	40.7 (5.5)	40.2 (28.8-61.7)	0	0	1	0.5
PROMIS Anxiety	36.3-82.7	203	50.2 (8.9)	51.3 (36.3-72.9)	34	16.8	0	0
PROMIS Depression	37.1-81.1	202	48.9 (8.9)	48.2 (37.1-77.9)	49	24.3	0	0
SF-36 PCS^a^	0-100	204	36.6 (9.1)	36.7 (14.0-59.8)	0	0	0	0
SF-36 MCS^a^	0-100	204	52.5 (9.2)	54.5 (21.6-68.1)	0	0	0	0
SF-36 Bodily Pain^a^	0-100	204	47.5 (18.6)	41.0 (0.0-100.0)	1	0.5	4	2.0
SF-36 Physical Function	0-100	204	52.0 (22.2)	50.0 (0.0-100.0)	2	1.0	3	1.5
WOMAC Pain	0-500	203	254 (98.6)	239.8 (50.7-500.0)	n/a	n/a	1	0.5
WOMAC Function	0-1700	204	899.0 (352.4)	906.7 (218.6, 1700.0)	0	0	1	0.5
6-min Walk Test^a^ ^b^ (meters)	n/a	199	395.5 (90.1)	392.8 (89.1-645.3)	n/a	n/a	n/a	n/a
20-Meter Walk Test^a^ (seconds)	n/a	202	19.0 (5.3)	18.2 (9.6-66.8)	n/a	n/a	n/a	n/a
PSS	0-40	203	13.3 (7.0)	13.0 (0.0, 37.0)	5	2.5	0	0
BDI-II	0-63	199	7.6 (8.6)	5.0 (0.0-39.0)	27	13.6	0	0

*PROMIS* = Patient Reported Outcomes Measurement Information System (reported as T-scores); *SF-36* = Medical Outcomes Short Form-36, *MCS* = Mental Component Summary, *PCS* = Physical Component Summary, *BP* = Bodily Pain; *WOMAC* = Western Ontario and McMaster Universities Arthritic Index; *PSS* = Perceived Stress Scale; *BDI-II* = Beck Depression Index II

^a^Higher scores indicate better health-related outcomes (for other measures, higher scores indicates worse health-related outcomes)

^b^Does not include patients who refused, or attempted and failed

### PROMIS pain interference

PROMIS Pain Interference demonstrated minimal floor effect with only 5 % of participants scoring the minimum PROMIS Pain Interference score. Pain Interference scores had stronger correlations with measures of whole body pain (SF-36 Bodily Pain, *r* = −0.73, 95 % confidence interval [CI] = −0.79 to −0.65) and physical health (SF-36 PCS, *r* = − 0.73, 95 % CI = −0.79 to −0.66) than other legacy measures, including knee-specific pain (WOMAC pain, *r* = 0.47, 95 % CI = 0.35 to 0.57) or gait speed (20-m walk test, *r* = 0.34, 95 % CI = 0.21 to 0.45.

### PROMIS physical function

PROMIS Physical Function scores had no floor effect since all of the participants scored above the minimum but one participant reported the highest possible score. PROMIS Physical Function scores had stronger correlations with measures of whole body function than with other legacy measures, including patient-reported lower extremity function or objective measures of lower extremity performance. Specifically, PROMIS Physical Function scores correlated well with SF-36 Physical Function (*r* = 0.79, 95 % CI = 0.73 to 0.84) compared with WOMAC function (*r* = −0.48, 95 % CI = −0.58 to −0.36), gait speed (*r* = −0.43, 95 % CI = −0.53 to −0.31), or 6-min walk times (*r* = 0.46, 95 % CI = 0.34 to 0.56).

### PROMIS anxiety

PROMIS Anxiety scores had a floor effect; 17 % of participants scored the minimum. For comparison, 3 % of participants had a minimum Perceived Stress Scale score. PROMIS Anxiety scores had stronger correlations with measures of stress (*r* = 0.71, 95 % CI = 0.64 to 0.77) and depression (*r* = 0.70, 95 % CI = 0.62 to 0.76) than other legacy measures. For example, PROMIS Anxiety scores had small to moderate correlations with measures of function (*r* = −0.40 to 0.17) and pain (*r* = −0.41 and 0.20).

### PROMIS depression

PROMIS Depression scores also had a floor effect; 24 % of participants scored the minimum. For comparison, 14 % of participants had a minimum BDI-II score. PROMIS Depression scores had stronger correlations with measures of stress (*r* = 0.65, 95 % CI = 0.56 to 0.72) and depression (*r* = 0.70, 95 % CI = 0.62 to 0.76) than with other legacy measures. For example, PROMIS Depression scores had small to moderate correlations with measures of physical function (*r* = −0.31 to 0.11) and pain (SF-36 Bodily Pain: *r* = −0.31 and WOMAC knee pain: *r* = 0.19).

### Comparison to previous literature

Figure 1 (PROMIS Pain Interference and Physical Function) and Fig. 2 (PROMIS Depression and Anxiety) visually locate the current study results in the range of findings from previous studies. In general, the values obtained in the current study were similar both to those obtained in other studies of individuals with osteoarthritis and in studies using other samples. As already noted, tests of the statistical significance of differences in associations were outside the scope of this study, and observed differences in point estimates should not be over-interpreted. We did note some putative differences that could warrant additional study (see Additional file 1: Table S1, Figs. 1 and 2). Pairs of measures that generated *lower* correlation estimates in the current study compared to previous studies included: a) PROMIS Pain Interference scores and SF-36 Bodily Pain scores, and b) PROMIS Physical Function scores with the WOMAC Pain, WOMAC Function, SF-36 Physical Function, and gait speed. Pairs of measures that generated *higher* correlation estimates in the current study compared to previous studies included: a) PROMIS Pain Interference scores and scores on patient-reported mental health scores (e.g., SF-36 MCS, BDI-II) and b) PROMIS Physical Function scores on the same measures.

## Discussion

PROMIS is a novel system of free instruments that clinicians and researchers in various medical disciplines can use. The PROMIS static short-forms offer an efficient and cost-effective means of measuring patient-reported outcomes critical to the assessment of knee osteoarthritis. This is the first study to evaluate the performance of the PROMIS static short-form instruments specifically among participants with symptomatic knee osteoarthritis. We found that PROMIS Anxiety and Depression measure a similar construct to other patient-reported outcomes that assess general mental health, including stress and depression, among patients with symptomatic knee osteoarthritis. The PROMIS Anxiety and Depression had a floor effect but the implications of this for future studies depend on study goals. For clinicians and researchers interested in distinguishing among individuals with low levels of anxiety or depression, the PROMIS short forms would not be a good choice. For example, if a researcher wished to explore an intervention’s impact on depression or anxiety by investigating whether it would benefit individuals with anxiety below the US mean then the short forms may not be ideal. The floor effect could impede detection of a treatment effect. However, these floor effects might be unimportant in a study of a clinical population in which the presence of anxiety and depression has been documented.

The associations we found between scores on PROMIS measures and legacy measures suggest that the PROMIS Pain Interference and Physical Function scores target whole body pain and physical function among individuals with symptomatic knee osteoarthritis. Recently, Broderick et al. demonstrated known-groups validity (a subtype of construct validity) of the computer adaptive administrations of PROMIS Pain Interference and Physical Function in a study comparing patients with osteoarthritis to the general population [27]. Our results complement and expand upon those of Broderick et al., specifically among individuals with symptomatic knee osteoarthritis. Our findings indicate that PROMIS Pain Interference and Physical Function have stronger correlations with assessments of whole-body disease burden (i.e., SF-36 Bodily Pain, SF-36 PCS) compared to joint-specific measures (i.e., WOMAC pain, WOMAC function, and physical function tests). Conceptually, this is expected since the two PROMIS static short-form instruments include questions that are not lower-extremity specific.

During the initial item-evaluation process of the full PROMIS Physical Function item bank, the data satisfied criteria for unidimensionality, suggesting that the four subdomains of mobility (lower extremity), dexterity (upper extremity), axial or central (neck or back), and complex activities could be collapsed into one domain [28]. Bruce et al. pointed out however, that the analysis was conducted among individuals with relatively little disease burden compared with the typical clinical trial participant, raising the question of whether the unidimensional model can be applied among other populations. After the data used in the current study were collected, Hays, et al. [29] identified upper-extremity and mobility subdomains of PROMIS Physical Function data. The two subdomains shared 35 % of the variance in common. Subsets of items targeting these two subdomains were identified and scored using PROMIS parameters. This work allows measurement of upper-extremity function and mobility as well as overall physical functioning within the PROMIS system. Clinicians and researchers, who are interested in the local effects of knee osteoarthritis, may prefer using PROMIS lower-extremity items [29] or a joint-specific pain and function outcome (e.g., WOMAC). However, if the goal is to assess whole body pain or function (e.g., assessing an exercise intervention among patients with osteoarthritis at the knee and other joints) then the short form may be appropriate. Ultimately, many investigators or clinicians may want to consider lower-extremity specific outcomes and whole body changes. Therefore, using a computer adaptive version of PROMIS or a larger set of the PROMIS items may enable the simultaneous assessment of whole-body and lower-extremity specific outcomes, which would reduce the number of outcome instruments a participants needs to complete and reduce study expenses associated with paying for multiple patient-reported outcomes.

Our study is limited. Our visual representation of correlation results from the current study and previous research highlighted some observable differences in point estimates of associations. It was outside the scope of this study to evaluate whether these putative differences were significantly different or accountable to random variation. First, because the order of outcome measure administration was consistent across participants, order bias and patient fatigue may have affected patients’ responses to self-report measures and performance on physical function tests. Second, our analyses did not include patients who refused or attempted and failed the two physical function tests (6-min walk test [*n* = 4]; 20-m walk test [*n* = 2]). Because patients who attempted and failed the physical function tests represent the extremes of functional impairment, our analysis overestimates the overall function of our cohort. However, this subgroup represents a small proportion of our overall sample and thus we did not feel this significantly influenced our overall results. Third, our definition of floor and ceiling effects was likely a conservative estimate. We measured the number of patients with the minimum and maximum scores rather than clinically-defined scores with thresholds representative of the extremes of health-related outcomes. Longitudinal studies will be needed to assess the minimally important change to better estimate the floor and ceiling effects.

More work needs to be done to understand the role of PROMIS static short-form instruments in evaluating patients with knee osteoarthritis. Future studies might assess time savings and cost efficiency, as well as other key psychometric properties as outlined by the Consensus-based Standards for the Selection of Health Measurement Instruments (COSMIN) [30]; including, responsiveness of scores on PROMIS short-forms in detecting change and score interpretability among patients with knee osteoarthritis. Lastly, computerized-adaptive administrations of PROMIS instruments should also be tested among patients with knee osteoarthritis.

## Conclusions

In conclusion, scores on the four tested PROMIS static short forms demonstrated good performance among patients with symptomatic knee osteoarthritis as evidenced in correlations with legacy measures. The patterns of correlations suggest that PROMIS Anxiety and Depression targets general mental health (e.g., stress, depression), and PROMIS Pain Interference and Physical Function static short forms target whole body outcomes (e.g., SF-36) among participants with symptomatic knee osteoarthritis. These instruments may be useful in clinical research among individuals with symptomatic knee osteoarthritis. However, floor effects in the PROMIS Anxiety and Depression scores should be considered if needing to distinguish among patients with very low levels of these outcomes.

## Additional file

Additional file 1:
**The additional file contains a supplemental table entitled “Matrix of Spearman correlation coefficients between PROMIS short-form instruments and legacy measures.”** The file also includes the raw data used for the figures and the appropriate references. (XLSX 48 kb)

## Abbreviations

PROMIS: Patient Reported Outcomes Measurement Information System
WOMAC: Western Ontario and McMaster Universities Arthritis Index
SF-36: Short form-36
BDI-II: Beck Depression Inventory Second Edition
PCS: Physical Component Summary
MCS: Mental Component Summary
